# Effects of dexmedetomidine and esmolol on systemic hemodynamics and exogenous lactate clearance in early experimental septic shock

**DOI:** 10.1186/s13054-016-1419-x

**Published:** 2016-08-02

**Authors:** Glenn Hernández, Pablo Tapia, Leyla Alegría, Dagoberto Soto, Cecilia Luengo, Jussara Gomez, Nicolas Jarufe, Pablo Achurra, Rolando Rebolledo, Alejandro Bruhn, Ricardo Castro, Eduardo Kattan, Gustavo Ospina-Tascón, Jan Bakker

**Affiliations:** 1 Departamento de Medicina Intensiva, Facultad de Medicina, Pontificia Universidad Católica de Chile, Marcoleta 367, Santiago, 8320000 Chile; 2 Unidad de Pacientes Críticos, Hospital Clínico Universidad de Chile Santos Dumont 999, Santiago, 8380000 Chile; 3 Universidade de Passo Fundo, Av. Brasil Leste, 285 - São José, Passo Fundo, RS 99052-900 Brazil; 4 Departamento de Cirugía Digestiva, Facultad de Medicina, Pontificia Universidad Católica de Chile, Marcoleta 367, Santiago, 8320000 Chile; 5 Intensive Care Medicine Department, Fundación Valle del Lili - Universidad ICESI, Avenida Simón Bolívar Carrera 98, Cali, 76001000 Colombia; 6 Department of Intensive Care Adults, Erasmus University Medical Center, PO Box 2040, Room H625, Rotterdam, CA 3000 The Netherlands

**Keywords:** Septic shock, Lactate, Lactate clearance, Dexmedetomidine, Esmolol

## Abstract

**Background:**

Persistent hyperlactatemia during septic shock is multifactorial. Hypoperfusion-related anaerobic production and adrenergic-driven aerobic generation together with impaired lactate clearance have been implicated. An excessive adrenergic response could contribute to persistent hyperlactatemia and adrenergic modulation might be beneficial. We assessed the effects of dexmedetomidine and esmolol on hemodynamics, lactate generation, and exogenous lactate clearance during endotoxin-induced septic shock.

**Methods:**

Eighteen anesthetized and mechanically ventilated sheep were subjected to a multimodal hemodynamic/perfusion assessment including hepatic and portal vein catheterizations, total hepatic blood flow, and muscle microdialysis. After monitoring, all received a bolus and continuous infusion of endotoxin. After 1 h they were volume resuscitated, and then randomized to endotoxin-control, endotoxin-dexmedetomidine (sequential doses of 0.5 and 1.0 μg/k/h) or endotoxin-esmolol (titrated to decrease basal heart rate by 20 %) groups. Samples were taken at four time points, and exogenous lactate clearance using an intravenous administration of sodium L-lactate (1 mmol/kg) was performed at the end of the experiments.

**Results:**

Dexmedetomidine and esmolol were hemodynamically well tolerated. The dexmedetomidine group exhibited lower epinephrine levels, but no difference in muscle lactate. Despite progressive hypotension in all groups, both dexmedetomidine and esmolol were associated with lower arterial and portal vein lactate levels. Exogenous lactate clearance was significantly higher in the dexmedetomidine and esmolol groups.

**Conclusions:**

Dexmedetomidine and esmolol were associated with lower arterial and portal lactate levels, and less impairment of exogenous lactate clearance in a model of septic shock. The use of dexmedetomidine and esmolol appears to be associated with beneficial effects on gut lactate generation and lactate clearance and exhibits no negative impact on systemic hemodynamics.

## Background

Persistent hyperlactatemia during septic shock resuscitation is associated with very high mortality and has been considered a hallmark of impending tissue hypoxia [1–8]. Therefore, some current guidelines recommend targeting resuscitation at normalizing lactate levels [4]. In clinical practice, this is accomplished by increasing cardiac output (CO) with fluid loading and/or inodilators [8, 9].

The paradigm of hypoxic-generated lactate has been recently challenged [1, 5]. Indeed, serum lactate levels during septic shock resuscitation represent a balance between aerobic or anaerobic generation, and clearance by different tissues. Consistent translational research has shown that muscle lactate generation can be triggered by epinephrine through β_2_-receptor stimulation, a process denominated aerobic glycolysis [10, 11]. On the other hand, an impaired lactate clearance might be present even without obvious signs of liver ischemia [12, 13]. In a recent experimental study, we found an early and severe impairment of exogenous lactate clearance to 10 % of sham values 1 hour after endotoxic [lipopolysaccharide (LPS)] shock induction [13]. This finding was not explained by liver hypoperfusion as tested with different techniques [13], although the pathogenic mechanisms were not explored. It is important to emphasize that in this study we addressed real clearance, applying a kinetic modeling after an intravenous (IV) bolus of sodium lactate that describes its elimination from the body over a short period of time [13]. The term “lactate clearance” has somehow been erroneously used in medical literature because a decrease in serum lactate levels could be caused either by a decreased production or increased clearance, and the inverse is also true [13, 14].

On the other hand, an overwhelming adrenergic response could contribute to persistent hyperlactatemia. Excessive α-mediated vasoconstriction might hasten hypoperfusion particularly in the hepatosplanchnic region, consequently increasing gut anaerobic lactate production, and impairing hepatic clearance by reducing portal or intrahepatic microcirculatory blood flow or eventually through metabolic effects [15–21]. High epinephrine levels can exacerbate aerobic glycolysis [10, 11]. The increasing awareness of the toxicity of sustained hyperadrenergia in critical illness has led to exploring adrenergic modulation or blockade as potential targets to attenuate adverse hemodynamic, microcirculatory, metabolic, and pro-inflammatory effects of sympathetic overstimulation [22–26]. Indeed, adrenergic modulation with an α_2_-agonist such as dexmedetomidine (DEX) [27–34], or β-blockers like esmolol (ESM) [35–40] have demonstrated favorable effects on diverse physiological and clinical outcome parameters in septic shock, and also on some potential determinants of persistent hyperlactatemia.

However, most of the studies with adrenergic modulation or β-blockade have been performed after the initial resuscitation period, and it is not known if these therapies are tolerated earlier in the evolution. In addition, since hyperlactatemia is a fundamental target for septic shock resuscitation, and due to the adrenergic influence over its determinants, it could be relevant to explore the impact of adrenergic modulation or β-blockade at this level. To address this subject, we performed a controlled experimental study in an endotoxic sheep model [13] aimed at (1) determining the effects of DEX and ESM as compared to LPS-control animals on lactate production and exogenous lactate clearance; and (2) evaluating the hemodynamic tolerance of DEX and ESM in the very early phase of septic shock. We hypothesized that both dexmedetomidine and esmolol decrease lactate production and attenuate impairment in exogenous lactate clearance in this model.

## Methods

The experimental design was performed in agreement with the Guide for the Care and Use of Laboratory Animals, 8th edition (2011), and with the approval of the Comité de Etica y Bienestar Animal of the Pontificia Universidad Católica de Chile (CEBA 12-031). We used a well-standardized model of LPS shock in sheep that induces a characteristic hyperdynamic profile [13]. Details of the experimental setup have been reported elsewhere and will be summarized below [13].

Our model addressed three of the major determinants of persistent hyperlactatemia: muscle lactate generation as potentially representing adrenergic-driven aerobic glycolysis through microdialysis and serum epinephrine levels; gut lactate production as potentially representing anaerobic generation through portal oxygen venous saturation and lactate levels; and clearance using the exogenous lactate clearance technique proposed by Levraut et al [12].

### Animal care and anesthesia

Sheep weighing 32 ± 5.2 kg were used. Animals were fasted 12 h before the experiments but with free access to water. Sheep were premedicated with 20 mg/kg ketamine and 0.25 mg/kg midazolam intramusculary. After inserting a peripheral intravenous line and injecting 30 mcg/kg fentanyl + 0.5 mg/kg atracurium + 1 mg/kg lidocaine intravenously, sheep were intubated and connected to mechanical ventilation in volume-control mode (Savina® 300, Dräger, Lübeck, Germany) with a tidal volume of 10 ml/kg. Anesthesia was sustained with a continuous infusion of midazolam, fentanyl, and ketamine. Muscle relaxation was maintained with a continuous atracurium infusion. During the surgical procedure, normal saline was infused at 10 ml/kg/h, and the rate was reduced to 5 ml/kg/h thereafter till the end of the experiment. Body temperature was kept at 38 ± 0.5 °C.

### Surgery and instrumentation

An 8-Fr sheath was placed in both the left and right external jugular veins to advance pulmonary artery and hepatic vein catheters, respectively. The latter were positioned under ultrasound guidance. The left femoral artery and vein were surgically exposed, and arterial and central venous catheters were introduced for blood pressure monitoring, sampling, and administering fluids and drugs.

The abdomen was opened via a midline laparotomy and gastric contents were drained through a small gastrostomy. After this, the splenic vein was ligated and a portal catheter was placed for sampling. An ultrasound flow probe (Transonic, Ithaca NY, USA) was positioned around the hepatic artery and the portal vein to measure total hepatic blood flow, and the laparotomy was closed. A microdialysis catheter was inserted in the anterior quadriceps muscle (Harvard Apparatus, Holliston MA, USA).

### Measurements

1. Hemodynamic data were recorded every 30 minutes: heart rate (HR), mean arterial pressure (MAP), pulse pressure variation (PPV), and pulmonary arterial and occlusion pressures were measured with the standard procedure and displayed on a multi-modular monitor (GE Healthcare, Datex-Ohmeda, Madison WI, USA). CO was assessed by thermodilution (CO module, GE Healthcare, Datex-Ohmeda, Madison WI, USA).
2. Systemic and hepatosplanchnic oxygen delivery and consumption: arterial, portal vein, hepatic vein, and mixed venous gases were assessed with a blood gas analyzer (i-Stat® bedside gas analyzer, Abbott Laboratories, Princeton NJ, USA). Total hepatic blood flow was measured with the ultrasound flow probe.
3. Lactate assessment: serum lactate levels at every experimental time point were obtained from arterial and venous blood samples, and measured directly with a lactate scout monitor (Senslab, Leipzig, Germany). Measurements were performed in triplicate and results were averaged.
4. Exogenous lactate clearance: lactate clearance was performed at the end of the experiment. An intravenous dose of sodium L-lactate (1 mmol/kg) was infused via the central venous catheter in 15 minutes [13]. Arterial blood samples were obtained at baseline and 1, 3, 6, 9, 15, and 20 minutes thereafter. Clearance was later calculated by the least squares technique with semi-logarithmic coordinates [13].
5. Muscle microdialysis was performed using a CMA/402 microinjection pump at 0.3 μL/min of perfusion flow rate coupled to the microdialysis probe CMA/20 (Harvard Apparatus, Holliston, MA, USA). A 1-h equilibration period was allowed after insertion of the probe. The dialysate was collected into sealed 250 μL glass tubes in a refrigerated (4 °C) fraction collector (CMA142). Dialysate fractions for lactate assessment were collected during the last 30 minutes before time points A to D (Fig. 1, see below).

**Fig. 1:**
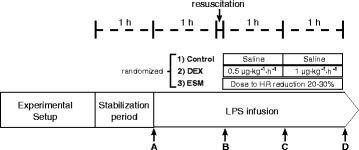
General scheme of the protocol. Complete hemodynamic, respiratory, and systemic and regional perfusion measurements were performed at points *A*, *B*, *C*, and *D*, except for lactate clearance that was performed at point *D. LPS* lipopolysaccharide, *DEX* dexmedetomidine, *ESM* esmolol, *HR* heart rate
6. Epinephrine levels: serum epinephrine levels were determined in duplicate with an enzyme immunoassay kit (Rock Mountain Diagnostics, Colorado Springs, CO, USA).

### Experimental protocol

After 1 h of postsurgical stabilization, basal measurements were performed (Fig. 1, point A). Then, septic shock was induced by administration of a 5 μg/kg LPS IV bolus (E coli 0111: β4; Millipore Sigma, St. Louis MO, USA) followed by a continuous LPS infusion at 4 μg/kg/h until the end of the experiment. During the first hour of LPS infusion no fluids or vasopressors were administered. Thereafter, resuscitation was performed with 5 ml/kg IV normal saline boluses repeated up to three times until a MAP of 55 to 60 mmHg or a pulse pressure variation below 10 % was achieved. If fluid loading failed to reestablish the MAP goal, norepinephrine (NE) was started at 0.1 mcg/kg/min and titrated in 0.1 mcg/kg/min increments every 5 minutes to the MAP target. Repeated series of measurements were performed after resuscitation (point B), and 1 and 2 h later (Fig. 1, points C and D, respectively). At each time point hemodynamic and respiratory variables, blood temperature, total hepatic blood flow, and arterial, mixed-venous, portal, and hepatic vein blood gases and lactate samples were taken simultaneously. Between points B and D, hypotension was basically managed with NE increments, but when the dose was increased by >0.5 mcg/kg/min during a 30-minute interval, and PPV was >15 % an additional 5 ml/kg fluid bolus was indicated.

After recording data set at point B, sheep were randomized into three groups of six animals each, LPS control, LPS-DEX (Precedex®, Hospira, Inc., Lake Forest, IL, USA), and LPS-ESM (Baxter Healthcare Corporation, Deerfield, IL, USA). To save animals and experiments we used only one LPS-control group against which DEX and ESM groups could be later compared. DEX was administered in two fixed doses for a 1-h period each (after point B, 0.5 μg/kg/h and after point C, 1.0 μ/kg/h). ESM was started at 15 mg/h and titrated every 5 minutes to achieve a reduction of 20–30 % in relation to HR at point B. This HR target was maintained until point D. Animals were euthanized with thiopental at the end of the experiment.

### Statistics

All data are presented as mean ± standard deviation (SD). Statistical testing was two-sided and used the 5 % significance level.

We calculated that six animals would be required per arm to detect a significant difference of 1.8 mmol/l in serum lactate levels, with a sigma of 1.0 mmol/l, between LPS-control and LPS-DEX or LPS-ESM groups, assuming a type I error rate of 5 % and a power of 80 %. This calculation was based upon data from a previous study in which after 150 minutes of evolution, LPS animals presented a mean lactate of 3.7 + 0.9 mmol/l as compared with 1.3 + 0.3 mmol/l in controls [41].

The Shapiro-Wilk test was used to test for normality, with *p* values > 0.10 indicating a normal distribution. Comparisons of different time points within a single group (A, B, C and D) were performed by using Friedman’s test with Bonferroni’s post hoc correction. Comparisons of continuous variables between two groups were conducted with the *t* test or the Mann-Whitney *U* test, as appropriate.

Since the main objective of our study was to explore the individual impact of two anti-adrenergic interventions on the main determinants of persistent hyperlactatemia, we only performed comparisons between LPS-control and LPS-DEX groups, and between LPS-control and LPS-ESM groups, but not between the study drugs. Statistical analysis was performed using STATA version 11.0 (StataCorp LP, College Station, TX, USA).

## Results

LPS induced a progressive septic shock with systemic hypotension, pulmonary hypertension and increasing NE requirements without differences between groups (Tables 1 and 2). DEX and ESM were well tolerated, and not associated with any adverse hemodynamic effect in terms of CO, mixed venous O_2_ saturation (SvO_2_), mixed venous-arterial carbon dioxide partial pressure gradients (p(v-a) CO_2_) or changes in NE requirements as compared to LPS controls (Tables 1 and 2). As expected LPS-ESM sheep exhibited a significant lower HR than LPS controls. The mean ESM dose was 26.7 ± 16.7 mg/h at point C and 32.2 ± 33.9 mg/h at point D.

**Table 1:** Comparison of hemodynamic variables between LPS-control and LPS-DEX group along the study period

Variable	Group	A	B	C	D	*p* ^a^
HR (bpm)	Control	127 ± 25	143 ± 16	137 ± 15	125 ± 20	
	DEX	139 ± 14	146 ± 27	128 ± 32	129 ± 30	
MAP (mmHg)	Control	88 ± 14	67 ± 14	59 ± 5	61 ± 7	a
	DEX	99 ± 20	63 ± 6	62 ± 12	58 ± 13	a
CO (ml/kg/min)	Control	78.3 ± 12.7	90.6 ± 26.4	80.0 ± 29.5	75.1 ± 23.1	
	DEX	89.0 ± 27.2	109 ± 21.2	92.1 ± 28.2	72.0 ± 25.9	
MPAP (mmHg)	Control	14 ± 2	20 ± 4	23 ± 9	23 ± 10	a
	DEX	13 ± 3	19 ± 7	21 ± 6	28 ± 5	a
PAOP (mmHg)	Control	8 ± 1	8 ± 1	7 ± 1	7 ± 1	
	DEX	9 ± 3	6 ± 2	7 ± 2	7 ± 3	
SvO_2_ (%)	Control	74 ± 4.5	78 ± 7.0	74 ± 10	69 ± 9.3	
	DEX	75 ± 5.5	77 ± 5.9	72 ± 5.9	68 ± 12	
P(v-a)CO_2_ (mmHg)	Control	4.7 ± 2.9	4.4 ± 3.6	6.9 ± 4.6	5.8 ± 4.0	
	DEX	4.1 ± 3.7	4.2 ± 1.8	3.5 ± 2.0	7.1 ± 1.1	
NE (μg/kg/min)	Control		0.77 ± 0.52	1.34 ± 0.6	1.88 ± 1.31	a
	DEX		1.08 ± 0.36	1.46 ± 0.38	1.62 ± 0.45	a

Values are presented as mean ± standard deviation (SD)

*LPS* lipopolysaccharide, *DEX* dexmedetomidine, *HR* heart rate, *MAP* mean arterial pressure, *CO* cardiac output, *MPAP* mean pulmonary arterial pressure, *PAOP* pulmonary artery occlusion pressure, *SvO*_*2*_ mixed venous oxygen saturation, *p(v-a)CO*_*2*_ mixed venous to arterial carbon dioxide partial pressure gradient, *NE* norepinephrine

*p* < 0.05 considered as significant

^**a**^Significant changes over time within groups (comparison made with Friedman test and post hoc Bonferroni correction)

**Table 2:** Comparison of hemodynamic variables between LPS-control and LPS-ESM groups along the study period

Variable	Group	A	B	C	D	*p* ^a^
HR (bpm)	Control	127 ± 25	143 ± 16	137 ± 15	125 ± 20	
	ESM	128 ± 19	133 ± 17	109 ± 12^*^	105 ± 9^*^	a
MAP (mmHg)	Control	88 ± 14	67 ± 14	59 ± 5	61 ± 7	a
	ESM	90 ± 18	70 ± 16	60 ± 8	76 ± 6	a
CO (ml/kg/min)	Control	78.3 ± 12.7	90.6 ± 26.4	80.0 ± 29.5	75.1 ± 23.1	
	ESM	69.0 ± 17	101 ± 32.0	84.0 ± 29.6	82.7 ± 28.4	
MPAP (mmHg)	Control	14 ± 2	20 ± 4	23 ± 9	23 ± 10	a
	ESM	16 ± 3	22 ± 7	22 ± 8	24 ± 7	a
PAOP (mmHg)	Control	8 ± 1	8 ± 1	7 ± 1	7 ± 1	
	ESM	7 ± 2	8 ± 2	8 ± 2	9 ± 2	
SvO_2_ (%)	Control	74 ± 4.5	78 ± 7.0	74 ± 10	69 ± 9.3	
	ESM	71 ± 5.1	73 ± 9	65 ± 11	65 ± 15	
P(v-a)CO_2_ (mmHg)	Control	4.7 ± 2.9	4.4 ± 3.6	6.9 ± 4.6	5.8 ± 4.0	
	ESM	5.2 ± 5.1	5.2 ± 3.2	7.2 ± 3.1	7.1 ± 4.0	
NE (μg/kg/min)	Control		0.77 ± 0.52	1.34 ± 0.6	1.88 ± 1.31	a
	ESM		0.87 ± 0.61	1.42 ± 0.61	1.52 ± 0.27	a

Values are presented as mean ± standard deviation (SD)

*LPS* lipopolysaccharide, *ESM* esmolol, *HR* heart rate, *MAP* mean arterial pressure, *CO* cardiac output, *MPAP* mean pulmonary arterial pressure, *PAOP* pulmonary artery occlusion pressure, *SvO*_*2*_ mixed venous oxygen saturation, *p(v-a)CO*_*2*_ mixed venous to arterial carbon dioxide partial pressure gradient, *NE* norepinephrine

*p* < 0.05 considered as significant

^**a**^Significant changes over time within groups (comparison made with Friedman test and post hoc Bonferroni correction)

^*^Significant difference between control group and ESM group, respectively at the same time point (comparison made with Mann-Whitney *U* test)

LPS induced a progressive hyperlactatemia in the three groups, although serum lactate levels at point C were lower with DEX and ESM as compared to controls. This effect was maintained for ESM at point D (Table 3). DEX group exhibited significant lower epinephrine levels as compared to controls at point D (Table 3). No difference in muscle lactate production between groups was observed.

**Table 3:** Evolution of serum lactate, muscle lactate, epinephrine levels, and exogenous lactate clearance at different time points

Control-DEX						
Variable	Group	A	B	C	D	*p* ^a^
Arterial lactate (mmol/L)	Control	2.0 ± 0.5	5.1 ± 1.8	8.1 ± 1.7	9.2 ± 1.8	a
	DEX	1.7 ± 0.5	3.4 ± 1.9	4.1 ± 1.9^*^	6.4 ± 3.1	a
Muscle lactate (mmol/L)	Control	3.8 ± 2.0	4.6 ± 1.3	5.1 ± 1.3	6.9 ± 3.5	a
	DEX	5.2 ± 4.4	4.9 ± 2.1	6.7 ± 3.6	6.7 ± 2.4	
Epinephrine levels (ng/ml)	Control	5.8 ± 4.3	5.6 ± 4.3	6.9 ± 4.2	7.3 ± 1.4	
	DEX	4.1 ± 1.8	3.2 ± 1.5	3.9 ± 3.6	4.6 ± 1.3^*^	
Lactate clearance (ml/kg/min)	Control				2.43 ± 1.14	
	DEX				6.97 ± 1.60^*^	
Control-ESM						
Variable	Group	A	B	C	D	*p* ^a^
Arterial lactate (mmol/L)	Control	2.0 ± 0.5	5.1 ± 1.8	8.1 ± 1.7	9.2 ± 1.8	a
	ESM	1.6 ± 0.4	3.7 ± 0.9	3.6 ± 1.0^*^	4.5 ± 1.1^*^	a
Muscle lactate (mmol/L)	Control	3.8 ± 2.0	4.6 ± 1.3	5.1 ± 1.3	6.9 ± 3.5	a
	ESM	3.3 ± 1.7	3.6 ± 3.2	5.0 ± 3.8	5.4 ± 2.6	
Epinephrine levels (ng/ml)	Control	5.8 ± 4.3	5.6 ± 4.3	6.9 ± 4.2	7.3 ± 1.4	
	ESM	4.8 ± 3.6	5.8 ± 7.1	9.0 ± 3.2	7.1 ± 2.4	
Lactate clearance (ml/kg/min)	Control				2.43 ± 1.14	
	ESM				7.32 ± 2.20^*^	

Values are presented as mean ± standard deviation (SD)

*DEX* dexmedetomidine, *ESM* esmolol

*p* < 0.05 considered as significant

^**a**^Significant changes over time within groups (comparison made with Friedman test and post hoc Bonferroni correction)

^*^Significant difference between control group and DEX or ESM groups, respectively at the same time point (comparison made with Mann-Whitney *U* test)

**Fig. 2:**
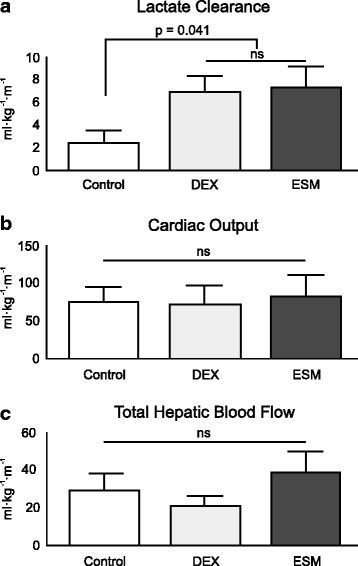
Comparison of dexmedetomidine, esmolol and controls in exogenous lactate clearance (**A**), cardiac output (**B**), and total hepatic blood flow (**C**) at the end of experiments. Both DEX and ESM were associated with less impairment in lactate clearance when compared to controls despite comparable systemic and regional hemodynamics. *DEX* dexmedetomidine, *ESM* esmolol

Portal lactate levels increased over time in all animals, but both DEX and ESM groups presented significant lower portal lactate values at points C and D as compared to LPS controls (Table 4). Exogenous lactate clearance was significantly higher in both DEX and ESM groups at point D (LPS-control 2.43 ± 1.14, LPS-DEX 6.97 ± 1.60, LPS-ESM 7.32 ± 2.2 ml/kg/min; *p* < 0.05) (Table 3 and Fig. 2). Total hepatic blood flow was comparable between groups (Table 4 and Fig. 2).

**Table 4:** Evolution of total hepatic blood flow and perfusion parameters at different time points

Control-DEX						
Variable	Group	A	B	C	D	*p* ^a^
SpO_2_ (%)	Control	81 ± 11	82 ± 7.1	78 ± 7.8	68 ± 11	a
	DEX	87 ± 6.0	85 ± 7.0	82 ± 8.3	78 ± 16	
ShO_2_ (%)	Control	75 ± 7.5	79 ± 5.2	72 ± 4.6	69 ± 14	
	DEX	76 ± 5.9	76 ± 6.1	74 ± 8.1	68 ± 17	a
Portal vein lactate (mmol/L)	Control	2.0 ± 0.5	4.2 ± 1.4	6.4 ± 1.0	8.1 ± 1.1	a
	DEX	1.7 ± 0.5	2.9 ± 1.5	3.7 ± 1.8^*^	6.2 ± 0.9^*^	a
Hepatic vein lactate (mmol/L)	Control	1.7 ± 0.5	4.4 ± 1.7	6.0 ± 1.1	7.3 ± 1.2	a
	DEX	1.4 ± 0.6	2.8 ± 1.6	3.4 ± 0.4^*^	5.8 ± 0.8^*^	a
Total hepatic blood flow (ml/kg/min)	Control	30 ± 8.8	36 ± 5.4	28 ± 7.7	25 ± 7.9	a
	DEX	27 ± 8.1	34 ± 5.6	23 ± 3.9	21 ± 4.9	
Control-ESM						
Variable	Group	A	B	C	D	*p* ^a^
SpO_2_ (%)	Control	81 ± 11	82 ± 7.1	78 ± 7.8	68 ± 11	a
	ESM	80 ± 6.1	79 ± 8.1	69 ± 12	75 ± 12	
ShO_2_ (%)	Control	75 ± 7.5	79 ± 5.2	72 ± 4.6	69 ± 14	
	ESM	70 ± 11	70 ± 16	68 ± 12	62 ± 23	
Portal vein lactate (mmol/L)	Control	2.0 ± 0.5	4.2 ± 1.4	6.4 ± 1.0	8.1 ± 1.1	a
	ESM	1.1 ± 0.4^*^	2.4 ± 0.7^*^	2.9 ± 0.9^*^	4.0 ± 1.1^*^	a
Hepatic vein lactate (mmol/L)	Control	1.7 ± 0.5	4.4 ± 1.7	6.0 ± 1.1	7.3 ± 1.2	a
	ESM	1.2 ± 0.4^*^	2.2 ± 0.6^*^	2.8 ± 1.0^*^	3.6 ± 1.4^*^	a
Total hepatic blood flow (ml/kg/min)	Control	30 ± 8.8	36 ± 5.4	28 ± 7.7	25 ± 7.9	a
	ESM	39 ± 8.0	37 ± 14	32 ± 13	28 ± 11	

Values are presented as mean ± standard deviation (SD)

*DEX* dexmedetomidine, *SpO*_*2*_ portal vein oxygen saturation, *ShO*_*2*_ hepatic vein oxygen saturation, *ESM* esmolol

*p* < 0.05 considered as significant

^**a**^Significant changes over time within groups (comparison made with Friedman test and post hoc Bonferroni correction)

^*^Significant difference between control group and DEX or ESM groups, respectively at the same time point (comparison made with Mann-Whitney *U* test)

## Discussion

The use of dexmedetomidine and esmolol was associated with lower arterial and portal lactate levels, and less impairment of exogenous lactate clearance in a model of septic shock. Both drugs were well tolerated when started very early after shock induction. DEX and ESM appear to be associated with beneficial effects on gut lactate generation and exogenous lactate clearance, and exhibit no negative impact on systemic hemodynamics.

Dexmedetomidine, an α_2_-agonist, attenuates sympathetic response to stress, and lowers epinephrine levels without adverse consequences on tissue perfusion [27–34]. Several experimental studies have consistently found anti-inflammatory effects and improvement in microcirculatory flow [32–34]. The drug is relatively well tolerated in anesthetized or critically ill patients, and even a post hoc analysis of the MENDS trial suggests an impact on mortality in septic patients [31]. Therefore, it could be useful as an adrenergic modulator in this setting.

On the other hand, the supporting evidence for β-blockers in sepsis is weak. Some small experimental and clinical studies have shown favorable effects on HR and hemodynamic or perfusion parameters, and also in inflammatory and metabolic parameters, particularly with nonselective blockers since most of these latter effects are β_2_ mediated [35]. However, β1 blockade could also exert anti-inflammatory effects, as was demonstrated by Hagiwara et al in a LPS rat model, on which an ultrashort-acting β-blocker inhibited nuclear factor-kappa B activity and attenuated histological lung damage [42]. Recently, a growing interest in esmolol, a short-acting selective β_1_-blocker has arisen mainly because of its pharmacokinetic characteristics [36–39]. An elegant experimental septic shock study found that ESM improves cardiac contractibility and vascular reactivity probably in relation to an anti-inflammatory effect [37]. In a randomized controlled study in stable septic shock patients, ESM reduced heart rate, decreased fluid requirements and lactate levels, and surprisingly showed a significant effect on mortality [38].

A disproportionate sympathetic response can be detrimental to critically ill patients as was demonstrated decades ago in another context such as chronic heart failure [22, 26]. Therefore, a growing interest in adrenergic modulation has arisen [22–26]. The big dilemma is to what extent can adrenergic modulation or blockade be accomplished without affecting basic survival responses especially in systemic hemodynamics. We found that both DEX and ESM appear to be well tolerated when started very early after shock onset, not only in terms of CO, MAP or NE requirements, but also from a metabolic point of view since both SvO_2_ and p(v-a)CO_2_ were comparable to LPS-controls. Furthermore, DEX and ESM were associated with favorable effects on both lactate generation and clearance as will be commented upon below. The few septic shock studies, in which ESM was assessed, started the drug hours after initial stabilization [38, 40]. In the case of DEX, this drug is not frequently used for primary sedation in septic shock patients due to the risk of inducing hemodynamic instability. DEX and clonidine might have opposite actions on vasomotor tone, a direct vasopressor, and indirect vasodilatory effects, with variable impact on MAP. When administered in healthy volunteers, DEX exerts a biphasic response, an initial increase in MAP due to stimulation of postsynaptic α_2b_ receptors followed by a long-lasting fall in MAP due to its central sympatholytic action with a decrease in epinephrine and NE blood levels [43]. Some investigators have tested the hypothesis that central sympaticolysis might help to restore adrenergic vasoconstrictor responsiveness in septic shock by reversing downregulation of alpha receptors secondary to high endogenous catecholamines, and some experimental data tend to support this as feasible [27, 44]. However, this effect might take longer time. In any case, the hemodynamic tolerance exhibited by both drugs in our study opens new opportunities for research in this relevant subject.

Septic shock triggers a strong compensatory sympathetic activation with a wide array of circulatory, metabolic and immune effects that could potentially impact lactate production or clearance [22, 26]. Among metabolic effects, epinephrine stimulates aerobic glycolysis in skeletal muscle cells through β_2_ stimulation. This process generates and releases lactate into the systemic circulation as a metabolic fuel [10, 11]. A dysregulated sympathetic stress response or exogenous catecholamines could also impair hepatosplanchnic or microcirculatory flow at the gut or the liver through excessive vasoconstriction, triggering anaerobic lactate generation and potentially impairing hepatic lactate clearance [15–21]. We designed our study to address three potential sources for persistent hyperlactatemia on which an overactive sympathetic response could exert some influence. DEX induced a 37 % reduction in serum epinephrine levels, but noteworthy, this was not associated to any negative effect, neither on hemodynamics, nor in muscle lactate outflow. Muscle lactate production can be decreased experimentally by different approaches and inversely, exogenous β_2_-adrenergic stimulation with epinephrine and other β_2_-agonists increases aerobic lactate generation [10, 11]. In this latter case, the threshold over which epinephrine might hasten muscle lactate outflow is unknown but clearly DEX in relatively high doses did not affect this process.

The effects on the hepatosplanchnic region are of particular interest. We observed that LPS animals treated with ESM and DEX exhibited less increase in portal lactate levels as compared with LPS controls. Additionally, portal venous O_2_ saturation decreased over time only in controls, whereas total hepatic blood flow tended to decrease in all groups. Progressive gut hypoperfusion eventually ameliorated by adrenergic modulation or blockade could explain these findings. Unfortunately, the study design does not allow us to affirm this with certainty since we did not measure mesenteric or mucosal microcirculatory flow directly. Clinical and experimental studies have yielded conflicting results on splanchnic lactate balance in sepsis [45–54]. While some studies report anaerobic lactate generation by the gut as regional flow decreases, other have minimized the contribution of gut-generated lactate to systemic hyperlactatemia, since most of this lactate would be normally cleared by the liver [45–54]. Nonetheless, if hepatic lactate clearance is simultaneously impaired, the systemic impact of gut-generated lactate might be higher.

In a previous study using the same model, LPS induced an early and severe impairment in exogenous whole body net lactate clearance that was not related to total liver hypoperfusion or evident biochemical dysfunction [13]. Indeed, the very low porto-hepatic vein lactate differences suggested at least a liver metabolic inability to handle increased lactate loads. The decrease in lactate clearance reached a 10 % of sham values at the end of the experiments [13]. In the present study, exogenous lactate clearance fell to extremely low levels in LPS-controls similarly than in our previous study, but this decrease was significantly attenuated both in DEX and ESM groups. The combined effects on gut perfusion and lactate clearance might explain the impact of DEX and ESM on serum lactate levels.

How can DEX and ESM actions decrease gut lactate generation or influence exogenous lactate clearance? We did not design this study as a mechanistic one, and therefore we can only speculate about the mechanisms. α_2-_agonists such as DEX can attenuate the sympathetic response to surgery, decreasing circulating catecholamine levels in at least 10 to 20 %, but in our LPS model it was almost 40 % [27, 28]. Interestingly, DEX might exert opposite actions on vasomotor tone, a direct vasopressor and indirect vasodilatory effects, with variable impact on MAP [27, 28]. However, some experimental studies have shown that α_2_ agonists could have predominantly favorable effects over the gut microcirculation [33, 34], a particularly vulnerable territory [55]. Yeh et al found that DEX prevented gut microcirculatory abnormalities induced by sympathetic activation after surgical stress in rats [33]. Miranda et al found a significant attenuation of capillary perfusion deficits with DEX in a LPS model [34]. Thus, it is possible that the favorable effect of DEX on portal lactate levels might be consequence of an attenuated adrenergic vasoconstriction on mesenteric or gut microcirculatory flow. It is more difficult to explain the effects of ESM since no direct vascular effect can be postulated. However, some experimental studies have shown protective vascular or microcirculatory effects potentially related to immunomodulation, or increased release of endothelial nitric oxide among other actions, but this should be confirmed by further research [35–38]. It is also well known that LPS can induce acute portal hypertension resulting in gut mucosal hypoperfusion [56] and that nonselective β-blockers might reduce portal hypertension, but this effect might not be extrapolated to β1-selective blockers. The favorable impact of DEX and ESM on exogenous lactate clearance can be hardly explained by hemodynamic effects, since only a small difference in total hepatic blood flow compared to controls was observed at the end of the experiments. Potential liver microcirculatory or cellular effects of DEX and ESM should be explored in future studies.

We acknowledge several limitations of our study. First, we did not assess directly gut or liver microcirculation, thus this precludes us establishing any conclusion on the microvascular effects of both drugs. Second, we did not evaluate immunological aspects or biomarkers, eventually missing the exploration of the impact of adrenergic modulation at this level in our model. Third, small differences in portal and hepatic vein lactate levels between ESM and controls were observed at baseline and after shock induction. Biological variability in response to surgical stress or LPS could explain this finding, but the strong differences still observed at points C and D support our conclusions. Fourth, since our study was not aimed at comparing DEX with ESM, but rather both drugs against LPS controls, we cannot formulate any conclusion concerning eventual superiority of one over the other. Finally, our study can be considered only as hypothesis-generating and therefore these results should be confirmed and expanded in further research.

## Conclusions

In conclusion, dexmedetomidine and esmolol were well tolerated and associated with lower arterial and portal lactate levels, and less impairment of exogenous lactate clearance in a model of endotoxic shock. Adrenergic modulation or blockade appears to be associated with beneficial effects on gut lactate generation and clearance, and exhibits no negative impact on systemic hemodynamics at least within the limits of our experimental model.

## Abbreviations

CO, cardiac output; DEX, dexmedetomidine; ESM, esmolol; HR, heart rate; IV, intravenous; LPS, lipopolysaccharide; MAP, mean arterial pressure; NE, norepinephrine; PPV, pulse pressure variation; p(v-a)CO_2_, venous-arterial carbon dioxide partial pressure gradient; SvO_2_, mixed venous oxygen saturation
